# Structural Insight into the Specific DNA Template Binding to DnaG primase in Bacteria

**DOI:** 10.1038/s41598-017-00767-8

**Published:** 2017-04-06

**Authors:** Yingqin Zhou, Hao Luo, Zhongchuan Liu, Mu Yang, Xiaoyun Pang, Fei Sun, Ganggang Wang

**Affiliations:** 1 Key Laboratory of Environmental and Applied Microbiology, Chengdu Institute of Biology, Chinese Academy of Sciences, Chengdu, 610041 China; 2 Key Laboratory of Environmental Microbiology of Sichuan Province, Chengdu, 610041 China; 3 National Laboratory of Biomacromolecules, CAS Center for Excellence in Biomacromolecules, Institute of Biophysics, Chinese Academy of Sciences, Beijing, 100101 China; 4 University of Chinese Academy of Sciences, Beijing, 100049 China

## Abstract

Bacterial primase initiates the repeated synthesis of short RNA primers that are extended by DNA polymerase to synthesize Okazaki fragments on the lagging strand at replication forks. It remains unclear how the enzyme recognizes specific initiation sites. In this study, the DnaG primase from *Bacillus subtilis* (*Bsu*DnaG) was characterized and the crystal structure of the RNA polymerase domain (RPD) was determined. Structural comparisons revealed that the tethered zinc binding domain plays an important role in the interactions between primase and specific template sequence. Structural and biochemical data defined the ssDNA template binding surface as an L shape, and a model for the template ssDNA binding to primase is proposed. The flexibility of the DnaG primases from *B*. *subtilis* and *G*. *stearothermophilus* were compared, and the results implied that the intrinsic flexibility of the primase may facilitate the interactions between primase and various partners in the replisome. These results shed light on the mechanism by which DnaG recognizes the specific initiation site.

## Introduction

The replication of a duplex DNA is a highly coordinated yet dynamic process that requires the assembly of multiprotein complexes to form a replisome^1^. At the DNA replication fork, the leading strand is synthesized continuously, whereas the lagging strand is made discontinuously^2^. Because DNA polymerases are incapable of initiating strand synthesis de novo, RNA primers are made by primase to prime DNA synthesis throughout replication; this priming activity is tightly coupled to the replisome by interactions with other replication partners^3, 4^.

In *Escherichia coli*, the primase (DnaG) transcribes ~2000 to 3000 RNA primers per replication cycle^5^. Biochemical studies have demonstrated that the DnaG primase initiates primer synthesis on specific template trinucleotides rather than a random sequence^6–8^. DnaG primase from *E*. *coli* catalyzes primer synthesis on 5′-d(CTG)^7, 9^ and DnaG primase in *Aquifex aeolicus* initiates preferentially on 5′-d(CCC)^10^. The DnaG primases of *Staphylococcus aureus*, *Geobacillus stearothermophilus*, *Bacillus anthracis* and *Bacillus subtilis* predominantly prime 5′-d(CTA)^11–14^. In T4 bacteriophage, the bacterial-like primase gp61 protein recognizes sequences containing 5′-d(GTT) and 5′-d(GCT) to initiate Okazaki fragments synthesis^15^, whereas T7 primase gp4 catalyzes primer synthesis on 5′-d(GTC)^8, 16^. Despite extensive efforts, the mechanism by which DnaG recognizes the specific initiation site has remained elusive.

The DnaG primase is conserved in all bacteria and consists of three functional domains, an N-terminal zinc binding domain (ZBD), a central RNA polymerase domain (RPD) and a C-terminal helicase binding domain (HBD)^17^. The crystal structures of the DnaG domains have been solved^18–20^. Additionally, based on the structure of RPD complexed with ssDNA, the translocation direction of the primase active site was defined^21^, however, this binding site of ssDNA is distant from the activity center. Recently, the structures of RPD/NTP complexes of *S*. *aureus* were determined and the predominant nucleotide-binding site of DnaG was revealed^22^. However, further efforts will be necessary to define the interactions between DnaG and specific ssDNA template.

To better understand the structure and function of primase and address the interactions between DnaG and specific DNA template, we have carried out structural and functional analysis on DnaG protein from *B*. *subtilis* (*Bsu*DnaG). The *Bsu*DnaG was overexpressed and crystallized, and the structure of RPD domain was determined. Biochemical data and structural comparisons revealed that the integrity of ZBD domain in primase is critical for DNA template binding and primer synthesis. Biochemical studies revealed that the template DNA may bind to the *Bsu*DnaG primase in L shape, the mechanism of primase recognizing the specific initiation site is discussed and a model of primase/ATP/ssDNA complex is proposed. These results provide valuable information for understanding the mechanism of RNA primer synthesis.

## Results and Discussion

### Overall structure

The purified *Bsu*DnaG was concentrated to 10 mg/ml for crystallization trials. The crystals belonged to space group P6_1_ with cell dimensions of *a* = *b* = 117.11 Å, *c* = 48.86 Å. There was one molecule per asymmetric unit. The RNA polymerase domain (RPD, residues 112–435) could be modeled in the electron density map. The final model of *Bsu*DnaG RPD has a *R*_*work*_ and *R*_*free*_ values of 18.9% and 23.9%, respectively, with an excellent geometry judged by the program MolProbity.

**Figure 1:**
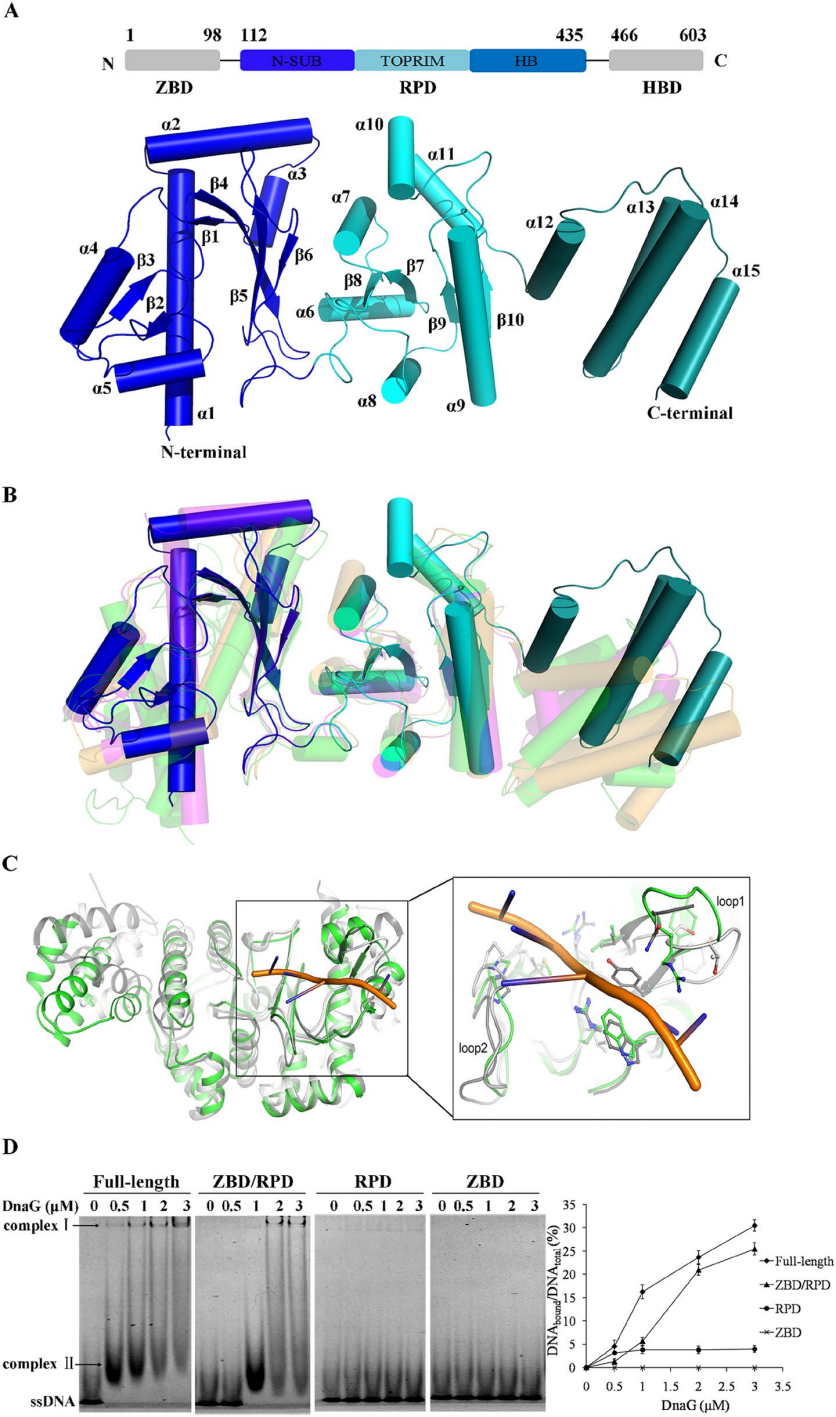
Structure of *Bsu*DnaG RPD. (**A**) RPD structure of *Bsu*DnaG. Secondary structural elements were numbered according to the primary sequence: α1-α15, β1-β10. (**B**) Structural comparison of the primase RPD between *B*. *subtilis*, *S*. *aureus* (in magenta, PDB ID: 4e2k), *E*. *coli* (in orange, PDB ID: 1dd9) and *A*. *aeolicus* (in gray, PDB ID: 2au3). (**C**) Structural comparison of the *B*. *subtilis* RPD (in green) and *A*. *aeolicus* ZBD/RPD (in gray, PDB ID: 2au3). (**D**) DNA binding assays of truncated fragments of *Bsu*DnaG. The reactions were carried out at protein concentrations of 0, 0.5, 1.0, 2.0 and 3.0 μΜ, respectively; the 23 mer S1 sequence (5′-CAGA(CA)_5_ CTA(CA)_3_-3′) (0.5 µM) was fluorescently labeled and used in the assays. The primase/ssDNA complexes are indicated with arrows. The complex I was stable, the complex II was not stable and in smear. The amount of complex I was quantified using ImageLab software (Bio-Rad).

The overall RPD structure of *BsuDnaG* displayed three subdomains, the N-terminal subdomain (residues 112–242, blue in Fig. 1A), the central TOPRIM subdomain (residues 243–367, cyan in Fig. 1A) and the C-terminal subdomain (residues 368–435, skyblue in Fig. 1A). Superposition of RPD structure of *B*. *subtilis* against that of *S*. *aureus*, *E*. *coli* and *A*. *aeolicus* led to r.m.s.d. values of 1.31 Å, 1.80 Å and 1.73 Å, respectively. Clearly, the structure of *BsuDnaG* RPD shared similar overall structures with its homologs, except for a slight variation in the orientation of the C-terminal helical bundle (Fig. 1B).

### DNA binding by *Bsu*DnaG

Structural comparison revealed that the orientation of loop1 in *Bsu*DnaG RPD was quite different from that in ZBD/RPD of *A*. *aeolicus*; it appeared that the deletion of ZBD resulted in loop1 rotated about 25 degrees anti-clockwise (Fig. 1C). In the ZBD/RPD structure of *A*. *aeolicus*, the ZBD domain was tethered to the RPD by hydrophobic interactions and salt bridges^23^. Sequence alignment revealed that the residues involved in the hydrophobic interaction were quite conserved in bacterial primase (Figure S1). Previous study showed that loop1 was involved in DNA binding^21^, thus, the deletion of ZBD may affect DNA binding to the RPD.

To test this hypothesis, three truncated fragments of *Bsu*DnaG including ZBD/RPD, RPD and ZBD were prepared for DNA binding assay. Previous study indicated *B*. *subtilis* DnaG primase preferentially initiates primer synthesis at 5′-CTA-3′ sequence in template^14^. A 23-mer oligonucleotide containing the CTA (S1 sequence) site was used in this binding assay. In gel-shift experiments (Fig. 1D), the full-length primase was observed to shift DNA completely at a protein concentration of 0.5 μM. The fragment of ZBD/RPD was able to shift all the DNA at a protein concentration of 1 μM. However, no stable RPD/ssDNA complex could be detected even at a protein concentration of 3 μM, and the ZBD didn’t bind to ssDNA at all. These results showed that ZBD/RPD still retained DNA binding activity, whereas the deletion of ZBD dramatically affected the DNA binding; the integrity of the ZBD/RPD is significant in ssDNA template binding. As described above, the deletion of ZBD led to a conformational change on loop1. Probably, there were also a few other delicate changes caused by the absence of ZBD fragment; the accumulation of these changes may lead to disturbance of the shape and positive charge network of the DNA binding surface. Eventually, the RPD will be null in template binding.

### The binding surface of the specific trinucleotide sequence

**Figure 2:**
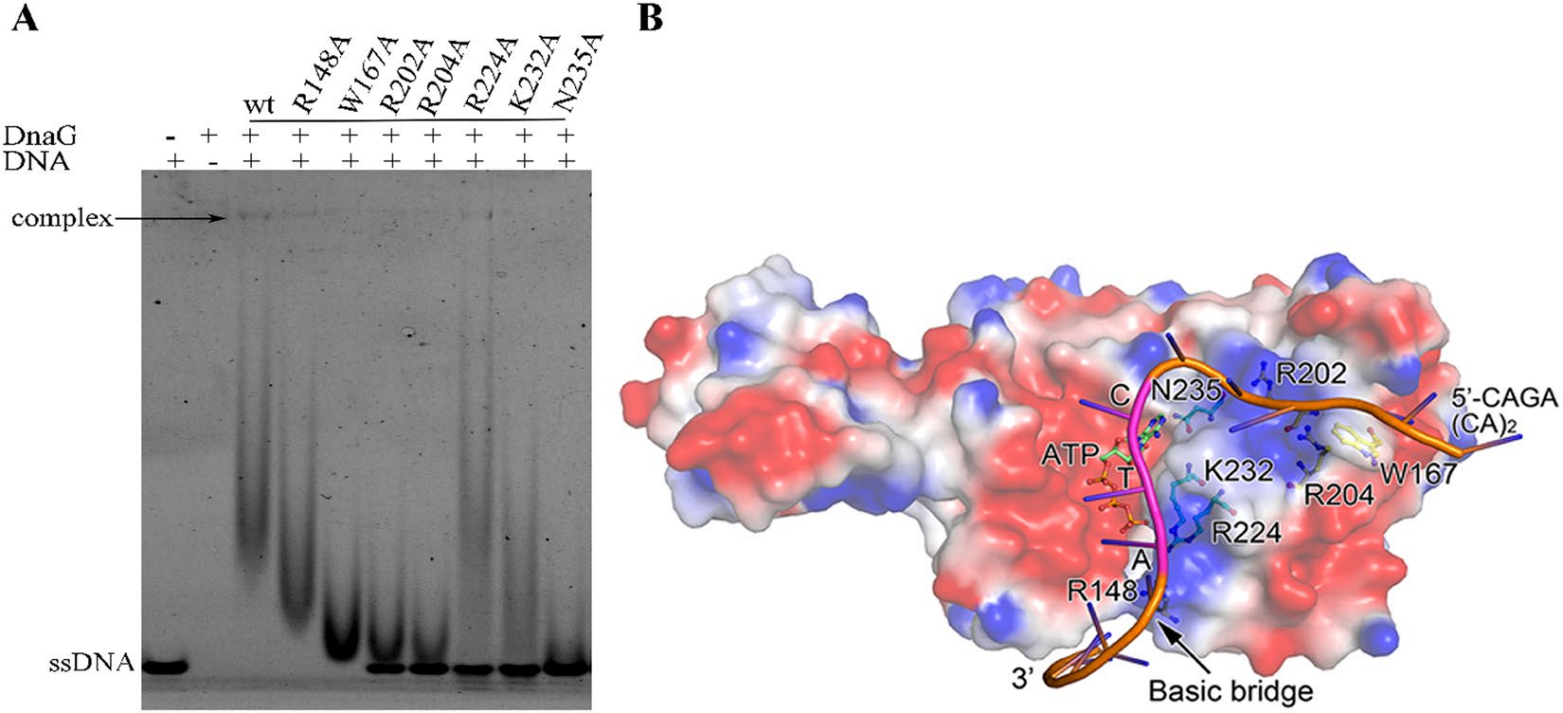
Interactions between DnaG Primase and ssDNA. (**A**) DNA binding assays of *Bsu*DnaG mutants. The concentration of DnaG proteins used in the experiment was 3.0 μΜ. The template ssDNA was the 23 mer S1 sequence. (**B**) A model of *Bsu*RPD/ssDNA/ATP complex. The model accommodates a 15 nt oligonucleotide in the binding site; the extra 8 nt oligonucleotide (5′-CAGA(CA)_2_-3′) is not shown in stick mode.

Since the S1 sequence used in the DNA binding assay contained a specific CTA site, we next asked where the specific DNA binding site is on the *Bsu*DnaG. One clue to resolve this question came from the crystal structures of *E*. *coli* RPD/ssDNA complex, the mutation of residues Trp165, Arg199, and Arg201 dramatically affected the DNA binding ability of RPD^21^. The counterparts (Trp167, Arg202 and Arg204) in *B*. *subtilis* were mutated, and the DNA binding activity of the mutants W167A, R202A and R204A was affected significantly (Fig. 2A). These results implied that the S1 sequence shares the binding site reported in the previous study^21^. In the structures of *S*. *aureus* RPD/NTP complexes, it was observed that the residues Arg146, Arg222, Lys230 and Asn233 interacted directly with NTP^22^. Sequence alignment revealed that these residues involved in NTP binding were conserved in bacterial primase. Here four mutants in *B*. *subtilis* (R148A, R224A, K232A and N235A) were prepared for DNA binding assay. The R148A mutant exhibited a slight reduction on DNA binding activity, in contrast, the mutants R224A and K232A showed more than 50% decrease in DNA binding activity, whereas N235A was of undetectable DNA binding activity (Fig. 2A). The results suggested that these residues were not only interacting with NTP but also involved in template DNA binding. Keck *et al*. proposed that the ssDNA threaded through the wedge-shaped cleft in the RPD domain, which was of positive electrostatic potential^18^. The study on the structure of RNP/ssDNA complex in *E*. *coli* redefined the binding geometry of the ssDNA template^21^. The results of mutagenesis studies here, in combination with the binding mode of NTP to the RPD^22^, suggest that the template bound to the primase is L-shaped (Fig. 2B). Previous studies showed that the DnaG primase synthesize an RNA primer beginning with the middle T residue at the sequence 5′-CTG-3′ and gives rise to a pppAG dinucleotide^8^. Thus, on the DNA template containing the initiation site CTA (S1 sequence), ATP will be the first nucleotide incorporated into the primer by the *B*. *subtilis* DnaG primase. So the T in CTA will be near to the tri-phosphate group of incorporated ATP to form a phosphodiester bond.

Overall, the binding mode of the S1 sequence can be defined based on information as follows: i) the results of the DNA binding activity of seven mutants which are involved in template DNA binding; ii) the structure of RPD/ssDNA complex in *E*. *coli* from which the geometry of the S1 sequence and partial binding site can be determined; iii) the structures of RPD/NTP complexes in *S*. *aureus* and the specific initiation site CTA from which the position of the CTA can be localized; iv) positive electrostatic potential of the binding site. In this model, the S1 template DNA binds to the primase in an L shape, a feature that has also been observed in other polymerases^24–27^. The 5′ end of the S1 sequence is near to the N terminus of the protein, and the phosphodiester bond will be formed between the ATP and the T in the CTA site (Fig. 2B).

Recognition of the initiation site by primase may depend on the fit between primase and the template DNA. A readout mechanism has been proposed for DNA recognition that emphasizes the connection between sequence and shape of DNA and the enrichment of arginines in the binding site of the protein; it is believed that a set of positive charges in the protein can recognize complementary shape of the DNA^28^. The primase may recognize the trinucleotide initiation site in the same way: the binding site functioning as a scanner and capturing the most appropriate template sequence to initiate priming. In each bacterium, the specific fit between primase and initiation site has been conserved due to structural or conformational constraints of primase and the specific DNA sequence.

### Proteolysis of DnaG primase

**Figure 3:**
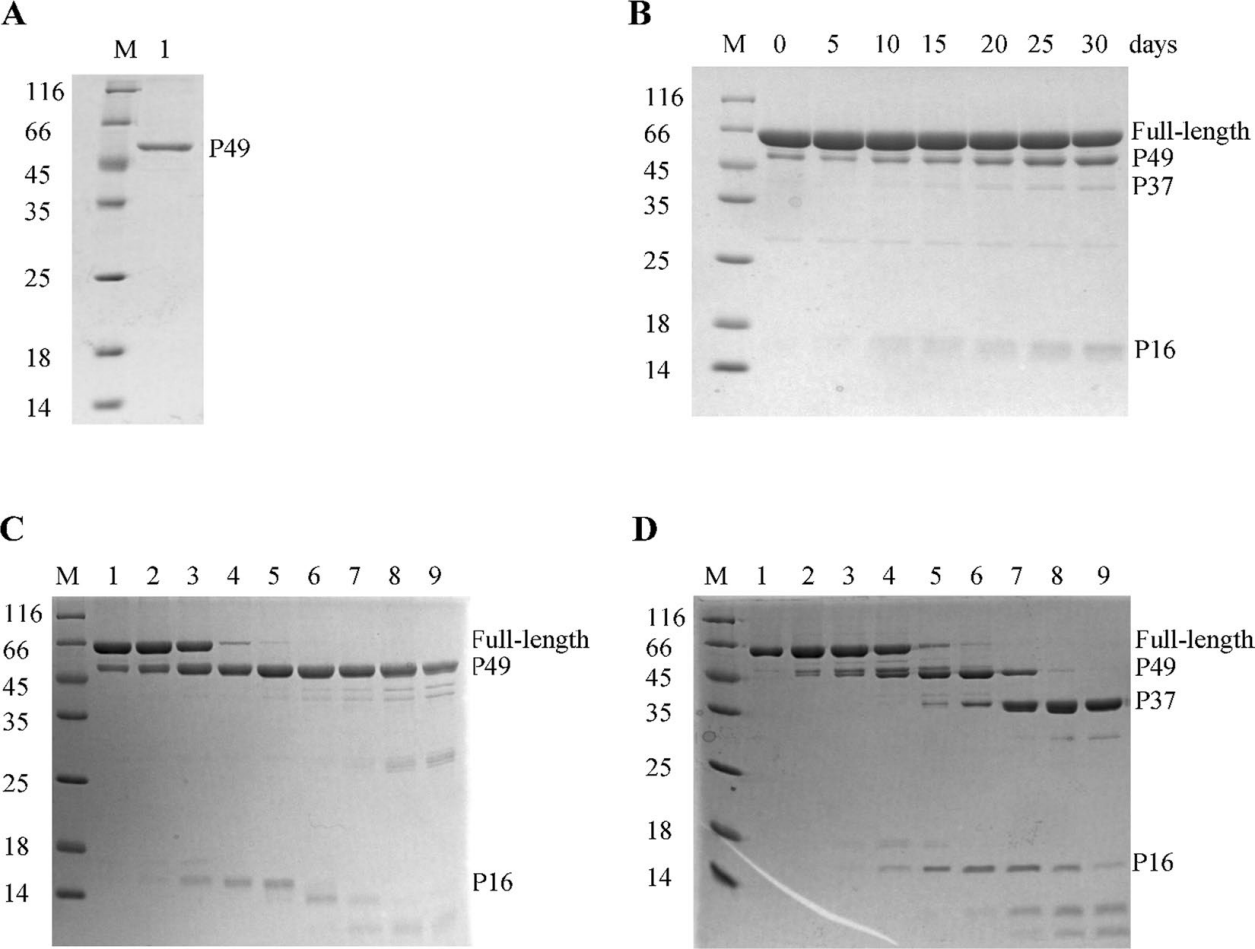
SDS-PAGE analysis on the degradation of *Bsu*DnaG primase. (**A**) SDS-PAGE analysis of dissolved crystals. (**B**) The *Bsu*DnaG protein was prone to degradation. The protein in solution was placed at 18 °C for different time (0, 5, 10, 15, 20, 25 and 30 days) and analyzed by 15% SDS-PAGE gel. (**C**) Trypsin digestion of DnaG primase from *B*. *subtilis*. (**D**) Trypsin digestion of DnaG primase from *G*. *stearothermophilus*. Two primases (10 µg) were digested under the same conditions, and the samples were analyzed by 15% SDS-PAGE. Lanes 1–9 represented the dosage of trypsin (0, 0.5, 1, 2.5, 5, 10, 25, 50 and 100 ng, respectively).

Full length *Bsu*DnaG was applied for crystallization trials and the structure of the RPD domain was solved. Thus, the crystals were harvested and dissolved for SDS-PAGE analysis (Fig. 3A). The results showed that proteolysis occurred in the crystallization drops and yielded a fragment of ~49 kDa. Additionally, the stability of *Bsu*DnaG protein in solution was studied at 18 °C. The results confirmed that *Bsu*DnaG gradually degraded itself (Fig. 3B).

The protein stability was also examined by trypsin digestion. The *Bsu*DnaG protein was incubated with various dosages of trypsin at room temperature for 30 min, and the progressive degradation products were then analyzed by SDS-PAGE (Fig. 3C). It was observed that *Bsu*DnaG was digested into two polypeptide fragments, 49 kDa and 16 kDa, by 5 ng trypsin. At high trypsin dosages, the P49 fragment was still the dominant product, while the P16 degraded into smaller pieces. Under the same conditions, the DnaG protein from *G*. *stearothermophilus* (*Gst*DnaG) was also treated by trypsin (Fig. 3D). In contrast to the *Bsu*DnaG, the *Gst*DnaG protein was digested into two fragments of 49 kDa and 16 kDa by 2.5 ng trypsin, and the P37 fragment was observed in the treatment with 5 ng of trypsin, which implied that the 49 kDa fragment was further digested to yield two fragments of 37 kDa and 12 kDa. With treatment with 50 ng trypsin, the P37 fragment was the dominant product, which was consistent with a previous report^29^. Two trypsin-sensitive sites have been identified in *Gst*DnaG^29^; one site is between K454 and K455 (site 1), thus generating the fragments of P49 and P16, the other one is between R105 and G106 (site 2), giving rise to P37 and P12 resulting from the cleavage of P49. Sequence alignment revealed that only site 1 was present in the *Bsu*DnaG (Figure S1), thus the digestion of *Bsu*DnaG by trypsin produced mainly the two fragments of (P49 and P16).

Up until now, all the crystal structures of DnaG domains have been resolved^18–20, 23^, whereas the intact structure of the enzyme have not yet been reported. The unsuccessful crystallization of full-length DnaG may primarily result from the intrinsic flexibility of the protein. Our results show that the DnaG primase could degrade at the sites in the hinge regions. However, this intrinsic flexibility of hinge region is essential to the function of DnaG primase. In DNA replication, the primase binds to the helicase by HBD domain, which is structural/functional homologous to the NTD of the DnaB helicase^30^; the ZBD/RPD is responsible for template binding and primer synthesis, moreover, the primase subsequently interacts with clamp loader for the synthesis of the complementary ssDNA by DNA polymerase^31^. In these dynamic processes, in which the primase is involved in complex interactions with DNA and other proteins of the replisome, the flexibility of the linker loop will facilitate the reorganization in the domains of the DnaG.

## Conclusions

In this study, we obtained the structure of *Bsu*DnaG RPD domain and addressed the interactions between *Bsu*DnaG and the specific template DNA. We found that the integrity of the ZBD/RPD is essential in ssDNA template binding, and the specific template DNA may bind to the primase in an L shape. To better define the chemical basis for primer initiation, elongation, and termination, further investigations are required to determine the ternary complexes of DnaG/template/primer.

## Materials and Methods

### Materials

Oligonucleotides used in this study were all synthesized by Sangon Inc (Shanghai, China). Column resins used for protein purification were purchased from GE Healthcare (USA). All other chemicals used for preparing buffers and solutions were reagent grade and purchased from Merck, Sigma-Aldrich and local suppliers. *Bacillus subtilis* 168, *Geobacillus stearothermophilus*, *E*. *coli* DH5α, *E*. *coli* BL21 (DE3) and pGEX-6P-1 were used for gene cloning.

### Protein expression and purification

The *dnaG* gene of *Bacillus subtilis* 168 (DSM 23778, DSMZ, Germany) was amplified by PCR from its genomic DNA with the 5′/3′ specific primers which introduced *BamH*I site and *Sal* I site, respectively. The PCR products were cloned into the vector of pGEX-6P-1, the gene sequence was confirmed by DNA sequencing. The recombinant plasmid was designated as pGEX-6p-1-*Bsu*DnaG. The plasmid was transformed into *E*. *coli* BL21 (DE3) and grown at 37 °C in LB medium containing 100 μg/ml ampicillin. When the OD_600_ reached about 0.4~0.6, 0.2 mM Isopropyl β-D-1-thiogalactopyranoside (IPTG) was added to induce protein expression for 16 h at 16 °C. The cells were harvested and resuspended in buffer A (25 mM Tris-HCl pH 8.0, 150 mM NaCl, 1 mM Dithiothreitol (DTT)) and lysed by sonication. The supernatant was collected by centrifugation for 30 min at 15,000 × g and purified with Glutathione Sepharose 4B affinity chromatography (GE Healthcare) equilibrated with buffer A. The fusion protein-bound beads were incubated with PreScission Protease at 4 °C overnight. The *Bsu*DnaG protein was eluted and further purified by the combination of Resource Q anion exchange column (GE Healthcare) Superdex 75 gel filtration column (GE Healthcare), and the protein fractions were pooled and concentrated using a centrifugal filter (Millipore). Finally, the purified protein was essentially homogeneous and >95% pure, as analyzed by SDS-PAGE.

All mutant *Bsu*DnaG proteins were generated according to the QuickChange mutagenesis protocol. These mutants were purified in the same way as described above for the wild-type protein. The truncated fragment P49 was obtained from the full-length protein using trypsin proteolysis. The truncated fragments of primase (P37 and P12) were expressed and purified as described previously^29^ Primase from *Geobacillus stearothermophilus* (*Gst*DnaG) was expressed and purified as previously described^32^.

### Crystallization, data collection and structure determination

Initial crystallization was carried out by hanging drop vapor diffusion at 18 °C, using the crystallization screen kits from Hampton Research. The purified *Bsu*DnaG was concentrated to 10 mg/ml in 25 mM Tris pH 8.0, 100 mM NaCl and 1 mM DTT for crystallization trials. A total of 1 μl protein solution was mixed with 1 μl well solution and equilibrated against 200 μl reservoir solution. Crystals were observed after two months in reservoir solution of 0.2 M sodium citrate tribasic dehydrate, 0.1 M Tris hydrochloride (pH 8.5) and 30% (w/v) polyethylene glycol 400. Before data collection, the crystals were cryoprotected by the addition of 20% (v/v) glycerol and flash frozen in liquid N_2_.

X-ray diffraction data of *Bsu*DnaG crystals were collected at 100 K using beam line BL17U at Shanghai Synchrotron Radiation Facilities (SSRF)^33^. Data sets were processed and scaled by HKL2000^34^. The phase and the initial model of *Bsu*DnaG were obtained by molecular replacement method using a polyalanine model of DnaG RPD domain (PDB 4e2k) from *Staphylococcus aureus*. The residues 116–363 and residues 367–428 were searched separately by using Phenix.AutoMR^35^. Coot^36^ and Phenix.refine^35^ were used for manually building and refinement, respectively. The qualities of the final models were checked with the program MolProbity^37^. Details of the overall refinement and final quality of the models were shown in Table 1. The program PyMOL (http://www.pymol.sourceforge.net/) was used to prepare structural figures.

**Table 1:** Data collection and refinement statistics.

Data collection and processing	
Crystal	DnaG (degradation) (res 112–435)
Synchrotron beam line	SSRF BL17U
Wavelength (Å)	0.97916
Space group	P6_1_
Unit-cell parameters	
a, b, c (Å)	117.11, 117.11, 48.86
Monomers per asymmetric unit	1
Resolution	29.3–2.50 (2.54–2.50)^a^
No. of unique reflections	13480 (677)^a^
Redundancy	13.3 (13.5)^a^
Completeness (%)	100 (100)^a^
Mean I/σ	20.7 (6.5)^a^
R_merge_ (%)	12.3 (42.9)^a^
Refinement statistics	
Reflections (working/test)	13069/606
R_work_/R_free_ (%)	18.9/23.9
Number of atoms	
Protein	2602
Water	76
Average B factor (Å^2^)	
Main Chain/Side Chain	34.5/37.2
Water	34.6
Ramachandran plot (%)	
Favoured	97.5
Allowed	2.5
R.m.s. deviations	
Bond lengths (Å)	0.008
Bond angles (°)	0.897

^a^The values in parenthesis means those for the highest resolution shell.

### DNA binding assays

DNA binding ability of *Bsu*DnaG primase was monitored using a gel electrophoretic mobility shift assay (EMSA). The DNA substrate (S1 sequence) used in binding assays was a single-stranded 23-mer oligonucleotide that contained the CTA initiation sequence (5′-CAGA(CA)_5_ CTA(CA)_3_-3′) and labeled at the 5′-end with 6-carboxyfluorescein (6-FAM). The assays were carried out in 20 µL reaction mixture containing 25 mM Tris-HCl pH 8.0, 100 mM NaCl, 10% (v/v) glycerol, 1 mM DTT, 5 mM MgCl_2_, 2 mM ATP, 0.5 µM ssDNA and a certain amount of DnaG proteins. The reactions were incubated at 37 °C for 30 min. Subsequently, samples were transferred onto ice and 2 µL loading buffer (25 mM Tris-HCl pH 8.0, 0.1 mM EDTA) was added. Finally the samples were analyzed by a non-denaturing 6% v/v polyacrylamide gel in 1 × TBE buffer. The gel was photographed by the Gel Doc XR + system (Bio-Rad).

### Limited proteolysis

Limited proteolysis on primase was carried out by using trypsin in 50 mM Tris pH 8.0, 100 mM NaCl, 5 mM MgCl_2_, 1 mM DTT and 10% v/v glycerol. The protein was digested with trypsin at different protein-to-protease ratios at ambient temperature for 30 min. The reactions were terminated by addition of PMSF to a final concentration of 2 mM, and SDS-PAGE loading buffer, followed by heating to 95 °C for 5 min. The samples were then loaded immediately onto a 15% SDS-PAGE for analysis.

## Electronic supplementary material

Supplementary information
