# Supplementary material for: Structural Insight into the Specific DNA Template Binding to DnaG primase in Bacteria
Source: Sci Rep. 2017 Apr 6;7:659. doi: 10.1038/s41598-017-00767-8 (PMC5429622; doi:10.1038/s41598-017-00767-8)
Supplement: Supplementary file 1 — Supplementary information [file 41598_2017_767_MOESM1_ESM.doc]

**Supplemental materials**

**Structural Insight into the Specific DNA Template Binding to DnaG primase in Bacteria**

Yingqin Zhou^1,2,4^, Hao Luo^1,2,4^, Zhongchuan Liu ^1,2^, Mu Yang^1,2,4^, Xiaoyun Pang^3^, Fei Sun^3^, Ganggang Wang ^1,2*^

^1^Key Laboratory of Environmental and Applied Microbiology, Chengdu Institute of Biology, Chinese Academy of Sciences, Chengdu, 610041, China;

^2^Key Laboratory of Environmental Microbiology of Sichuan Province, Chengdu, 610041, China;

^3^National Laboratory of Biomacromolecules, CAS Center for Excellence in Biomacromolecules, Institute of Biophysics, Chinese Academy of Sciences, Beijing, 100101, China;

^4^University of Chinese Academy of Sciences, Beijing, 100049, China.

*Corresponding author:

SEND CORRESPONDENCE TO*:*

Ganggang Wang

Key Laboratory of Environmental and Applied Microbiology, Chengdu Institute of Biology, Chinese Academy of Sciences, Chengdu, 610041, China

Tel: 86-28-82890828; E-mail: [wanggg@cib.ac.cn](mailto:wanggg@cib.ac.cn)

**Supplemental Figures**


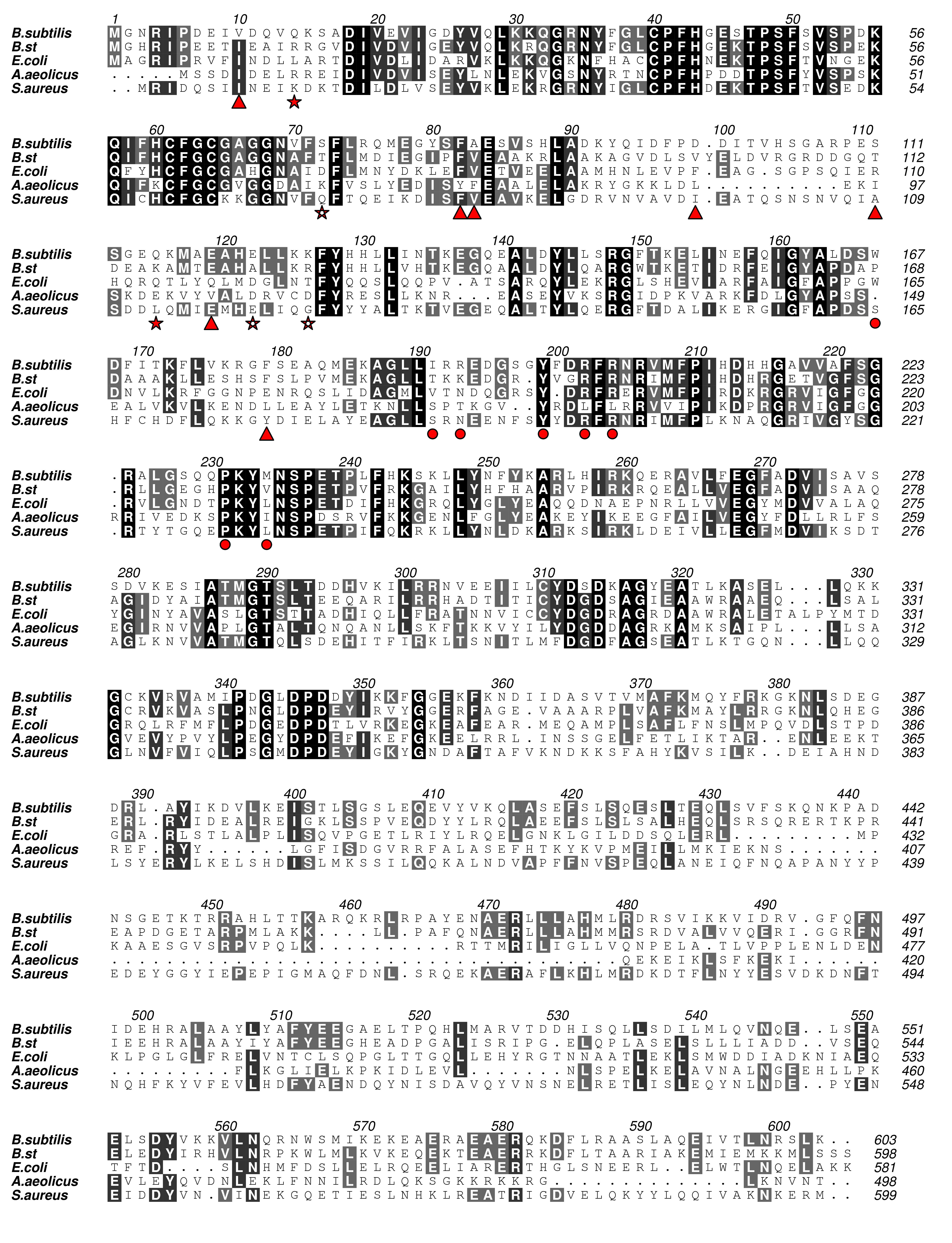


**Figure S1. Amino acid sequence alignment of the DnaG primase.** DnaG primase from *B. subtilis*, *G. stearothermophilus*, *S. aureus*, *A. aeolicus* and *E. coli* were aligned. The ZBD tethered to the RPD by hydrophobic interaction and salt bridges from the ZBD/RPD structure of *A. aeolicus*. The residues involved in the hydrophobic interaction and salt bridges were quite conserved among these bacterium. The triangles and the stars denoted the residues involved in hydrophobic interactions and salt bridges between ZBD and RPD domains, respectively. The solid spheres represented the residues involved in the DNA binding in the structure of *E. coli* RPD/ssDNA complex (PDB ID: 3b39).
